# Discrete choice experiment to determine preferences of decision-makers in healthcare for different formats of rapid reviews

**DOI:** 10.1186/s13643-021-01647-z

**Published:** 2021-04-20

**Authors:** Christian Speckemeier, Laura Krabbe, Susanne Schwenke, Jürgen Wasem, Barbara Buchberger, Silke Neusser

**Affiliations:** 1 Institute for Healthcare Management and Research, University of Duisburg-Essen, Thea-Leymann-Str. 9, 45127 Essen, Germany; 2 Scossis, Karmeliterweg 42, 13465 Berlin, Germany; 3 Robert Koch Institute, Nordufer 20, 13353 Berlin, Germany

**Keywords:** Evidence synthesis, Rapid review, Preference, Discrete choice experiment, Conjoint analysis

## Abstract

**Background:**

Time-saving formats of evidence syntheses have been developed to fulfill healthcare policymakers’ demands for timely evidence-based information. A discrete choice experiment (DCE) with decision-makers and people involved in the preparation of evidence syntheses was undertaken to elicit preferences for methodological shortcuts in the conduct of abbreviated reviews.

**Methods:**

D-efficient scenarios, each containing 14 pairwise comparisons, were designed for the DCE: the development of an evidence synthesis in 20 working days (scenario 1) and 12 months (scenario 2), respectively. Six attributes (number of databases, number of reviewers during screening, publication period, number of reviewers during data extraction, full-text analysis, types of HTA domains) with 2 to 3 levels each were defined. These were presented to the target population in an online survey. The relative importance of the individual attributes was determined using logistic regression models.

**Results:**

Scenario 1 was completed by 36 participants and scenario 2 by 26 participants. The linearity assumption was confirmed by the full model. In both scenarios, the linear difference model showed a preference for higher levels for “number of reviewers during data extraction”, followed by “number of reviewers during screening” and “full-text analysis”. Subgroup analyses showed that preferences were influenced by participation in the preparation of evidence syntheses.

**Conclusion:**

The surveyed persons expressed preferences for quality standards in the process of literature screening and data extraction.

**Supplementary Information:**

The online version contains supplementary material available at 10.1186/s13643-021-01647-z.

## Background

Healthcare policymakers require evidence-based information for their decision-making processes [1]. Health technology assessments (HTAs) provide this information and are typically based on a systematic review of the best available evidence. Due to the high level of methodological rigor [2], the preparation of well-conducted systematic reviews is a time-consuming task. It often takes between six months and one year until a systematic review is finalized and more than a year to complete a HTA report [3]. However, evidence to support urgent and emergent decisions related to procurement, clinical practice, and policy is often needed in a short period of time [4]. According to the European Transparency Directive (Directive 89/105/ EEC), relative effectiveness assessments need to be performed in a limited timeframe (90 days for pricing or reimbursement decisions or 180 days for pricing and reimbursement decisions) in order to achieve fast access for patients to medicinal products [5]. Another example would be the case of global public health crises, such as the COVID-19 pandemic, when up to date summaries of important information are needed in a limited timeframe [6].

New and abbreviated formats of evidence syntheses are up for discussion which are conducted within shorter timeframes and may be less expensive [3]. Frequent terms describing these formats are “rapid review”, “rapid evidence assessment,” “rapid systematic review,” or “rapid health technology assessment” (hereinafter, the common term “rapid review” is used) [7]. By now, different types of rapid review products exist which employ a broad range of strategies to alter the standard systematic review methods, with respect to purpose, methods, extent, resources, and timeframes [1, 8]. However, while the term “rapid” implies time savings, there is currently no consensus on how to realize these time savings and thus, no standardized methodology for conducting rapid reviews [7–11]. Although a number of guidelines have been published to support the conduct of rapid reviews, few of them offer a rationale for the recommended shortcuts [7–12]. In addition, many rapid reviews do not describe the methodology applied [7, 12]. Lately, the emergence of COVID-19 has led to an explosion of rapid reviews and initiatives to support rapid reviewers, such as a dedicated website of the Cochrane Collaboration entitled “Rapid Reviews in response to COVID-19", a fast-tracking of PROSPERO registrations, and free access to Covidence software for researchers concerned with COVID-19 [6].

While rapid reviews aim to fulfill the demands of healthcare decision-makers in a timely manner, the resulting methodological shortcuts bear the risk that results may be less reliable than those of systematic reviews [13]. Despite these limitations, decision-makers have high expectations regarding the validity of rapid reviews. Research has shown that decision-makers in healthcare and guideline developers expect rapid reviews to provide answers similar to systematic reviews in at least nine out of 10 cases [14]. Because of this apparent incompatibility, it was deemed important to investigate suitable formats of rapid reviews from the viewpoint of decision-makers and people involved in the preparation of evidence syntheses. In this study, different decision scenarios are presented to decision-makers in healthcare and researchers preparing evidence syntheses in order to determine preferences for methodological shortcuts in the development of rapid reviews. A common method to elicit preferences are discrete choice experiments (DCE). This technique is based on the assumption that any good or service can be described by its constituting characteristics (hereinafter called “attributes”) and that the extent to which an individual values a good or service is determined by the levels of these attributes [15]. In DCEs, respondents are presented different stimuli consisting of attributes with different levels and asked to state their preferences. The holistic assessments are then traced back to the contributions of the individual characteristics [16]. The method offers the advantage of a realistic assessment situation, as it specifically identifies respondents’ trade-offs when choosing goods or services [17]. DCEs are sensitive to changing levels of input and thus enable respondents to prioritize differing degrees of input, allowing trade-offs among choices [18].

One of the challenges in DCEs is their complexity related to the number of tasks. In so-called full factorial designs, combinations of all attribute levels are used. In practice, these designs are often not feasible as the number of evaluations required from each respondent becomes prohibitively large. For example, an experimental design consisting of six attributes with three levels each would result in *n*=3^6^=729 possible combinations. To deal with this problem, optimal designs can be used in which, based on certain quality criteria, an appropriate subset (fractional design) is selected from the set of theoretically possible stimuli. Thus, a reduced number of comparisons is required [19]. The present study aims to elicit preferences for methodological shortcuts in the conduct of rapid reviews by conducting a DCE directed at decision-makers in healthcare and researchers preparing evidence syntheses.

## Materials and methods

### Preliminary work

A key stage in the conduct of this study was to ensure that relevant attributes and levels were included in the DCE. Therefore, websites of organizations producing abbreviated evidence syntheses were searched for methodological guidance of rapid reviews. A list of organizations is shown in Sup. Table 1. Methodological guidance was included if (i) the document included methodological shortcuts compared to general systematic review methods, (ii) the guidance was marked as valid at the time it was identified, and (iii) the authors state that the approach is suitable for general application (i.e., not restricted to specific topics). In order to generate a broad information basis, no restrictions were applied regarding the publication period. Based on the results, formats of rapid reviews and key components were identified. An expert panel was conducted to discuss the relative importance and options for scaling. The consultation meeting consisted of experts from the Institute for Healthcare Management and Research and two external methodological experts involved in research on the development of rapid reviews. After two pretest runs, the key components were merged into six attributes by combining partial aspects to meaningful superordinate attributes in order to improve the feasibility of the survey. The attributes do not overlap and thus are independent.

### Participant recruitment

A sample of decision-makers in healthcare and people involved in the preparation of evidence syntheses was generated via the network of contacts of the Institute for Healthcare Management and Research. Eligible persons were contacted by e-mail and asked to participate in the online survey. A three-wave e-mail schedule was followed. First, an announcement e-mail was sent to 204 eligible individuals in June 2019, containing general information on the aim and purpose of the survey. A week later, the individuals received an e-mail containing a link to the web-based survey. Three weeks later, a follow-up e-mail was sent and after five weeks, the survey was deactivated. The web-based survey was accessible on the QuestionPro Survey (San Francisco, CA, USA) platform. The questionnaire contained questions on age, sex, company affiliation, managerial responsibility, and usage of evidence syntheses. The participants were then asked to complete the pairwise comparisons. Finally, the participants were asked to rate the importance of 11 aspects of an evidence synthesis on a five-point scale (1: very unimportant, 5: very important).

### Sample size

Currently, no standard exists for the determination of the minimum sample size in DCEs. Johnson and Orme have recommended a rule-of-thumb: nta/c ≥ 500, where *n* = number of respondents, *t* = number of tasks, *a* = number of alternatives per task, and *c* = the largest number of levels for any one attribute [18]. In the present study, this formula results in a minimum number of 54 respondents. However, as the general relevance of the attributes rather than the exact estimate were of importance in our analysis, also a smaller sample size was assumed to be sufficient.

### Definition of scenarios

Two scenarios on the basis of German regulations were defined, namely: (i) It has to be evaluated if a telemedical service should be implemented as a new medical examination and treatment method. Therefore, a rapid review has to be elaborated within 20 working days. (ii) The necessity, efficiency, and expediency of laboratory and human genetic services in the outpatient sector have to be evaluated. Within 12 months, a rapid review has to be elaborated. D-efficient choice sets were developed within QuestionPro Survey Software. As the scenarios address different timeframes for the conduct of rapid reviews, the number of levels assigned to the attributes was different. Therefore, distinct choice sets had to be developed (see Table 1, Sup. Table 2). The participants were randomized into two groups and each participant received a link to a version of the survey containing one of the two scenarios. Due to the complexity of the overall topic and according to the ISPOR Good Research Practices for Conjoint Analysis Task Force, which recommends the number of comparisons to be between eight and 16 [19], the number of pairwise choice tasks per participant was set at 14.

**Table 1:** Attributes and levels for scenario 1

Attribute		Level 1	Level 2	Level 3
**Database searches**	Number of databases	Medline or another database	Medline + 1 further database	Medline + 2 further databases
	Number of reviewers for screening	1 reviewer	2 reviewers, no seeking for consensus	2 reviewers and seeking for consensus
	Publication period to be considered	Last 2 years	Last 5 years	Last 10 years
**Data extraction**	Number of reviewers for data extraction	1 reviewer, no quality assurance	2 reviewers, no quality assurance	2 reviewers and quality assurance
	Full-text analysis	No full-text analysis	Full-text analysis for easily obtainable literature only	Full-text analysis
**Extent**	Type of HTA-domains	Safety, efficacy	Safety, efficacy, economic aspects	Safety, efficacy, economic, and further aspects

### Definition of attributes and levels

Level characteristics were derived from the identified rapid review formats. Two or three levels were defined for each attribute, with higher levels representing a stronger expression of the attribute. For example, the attribute “full-text analysis” in scenario 1 is made up of the three levels (i) no full-text analysis, (ii) full-text analysis only for easily obtainable literature, and (iii) full-text analysis. The individual levels are mutually exclusive. An overview of the attributes and their levels for scenarios 1 and 2 is shown in Table 1 and Supplementary Table 2 respectively. An exemplary choice task is shown in Sup. Figure 1.

### Statistical analysis

Descriptive statistics were used to summarize demographic characteristics of the first five survey questions. Responses to the DCE were analyzed using logistic regression models in SAS 9.4 (SAS Institute Inc. Cary, NC, USA). Full models were used to check the linearity of the linear difference model. In full models, no linear order is presupposed and thus, they were applied to investigate whether the order of levels corresponds to their predefined order and whether the distances between levels can be regarded as uniform. If this was the case, the further analysis was based on linear difference models. These parsimonious models were used to assess the relative importance of the individual attributes assuming uniform distances between levels. The Wald test was used to test the statistical significance of the individual regression coefficients. Additionally, descriptive subgroup analyses were performed by repeating the main analysis for a selected population. Due to an expected small sample size, the importance of the respective subgroup was not assessed (e.g. if age is a factor which significantly influences decisions, in the sense that younger participants make decisions based on different factors than older participants). Consequently, the analysis was carried out in an exploratory manner and therefore has to be interpreted with caution. The regression coefficients and odds ratios (OR) with their respective 95% confidence intervals (CI) are reported.

The descriptive analysis of the last question dealing with 11 aspects of an evidence synthesis was performed in SPSS (IBM Corp. Armonk, NY, USA). As this question deals with general characteristics of evidence synthesis and is not related to scenario 1 or 2, a combined analysis was undertaken.

## Results

A total of 62 persons participated and completed a total of 868 pairwise comparisons. Of these, 36 persons participated in scenario 1 and 26 persons participated in scenario 2. The response rate was 30.4%. The participants needed 15 minutes to complete the survey, on average. Participants’ characteristics are shown in Table 2.

**Table 2:** Characteristics of participants

Measure		Scenario 1, ***n*** = 36 (%)	Scenario 2, ***n*** = 26 (%)	Overall, ***n*** = 62 (%)
Sex	Male	21 (58.3%)	18 (69.2%)	39 (62.9%)
	Female	12 (33.3%)	7 (26.9%)	19 (30.6%)
	Missing	3 (8.3%)	1 (3.8%)	4 (6.4%)
Age category, years	18–24	0 (0%)	0 (0%)	0 (0%)
	25–34	0 (0%)	3 (11.5%)	3 (4.8%)
	35–44	5 (13.9%)	5 (19.2%)	10 (16.1%)
	45–54	15 (41.7%)	6 (23.1%)	21 (33.9%)
	55–64	13 (36.1%)	11 (42.3%)	24 (38.7%)
	> 65	0 (0%)	1 (3.8%)	1 (1.6%)
	Missing	3 (8.3%)	0 (0%)	3 (4.8%)
Affiliation	Statutory health insurance	8 (22.2%)	1 (3.8%)	9 (14.5%)
	National Association of Statutory Health Insurance Physicians (Kassenärztliche Bundesvereinigung)	1 (2.8%)	4 (15.4%)	5 (8.1%)
	Private health insurance	2 (5.6%)	3 (11.5%)	5 (8.1%)
	Medical service of health insurance agencies (Medizinischer Dienst der Krankenkassen)	0 (0%)	3 (11.5%)	3 (4.8%)
	Other	4 (11.1%)	7 (26.9%)	11 (17.7%)
	Missing	21 (58.3%)	8 (30.8%)	29 (46.8%)
Managerial responsibility	No	9 (25.0%)	8 (30.8%)	17 (27.4%)
	≤ 10 employees	9 (25.0%)	8 (30.8%)	17 (27.4%)
	11–25 employees	6 (16.7%)	3 (11.5%)	9 (14.5%)
	26–50 employees	1 (2.8%)	3 (11.5%)	4 (6.5%)
	> 50 employees	8 (22.2%)	4 (15.4%)	12 (19.4%)
	Missing	3 (8.3%)	0 (0%)	3 (4.8%)
Usage of evidence syntheses	I use evidence syntheses to inform myself	4 (11.1%)	1 (3.8%)	5 (8.1%)
	I use evidence syntheses to inform myself and others	18 (50.0%)	11 (42.3%)	29 (46.8%)
	I use evidence syntheses to make decisions	5 (13.9%)	4 (15.4%)	9 (14.5%)
	I am involved in the preparation of evidence syntheses	4 (11.1%)	8 (30.8%)	12 (19.4%)
	Other	1 (2.8%)	1 (3.8%)	2 (3.2%)
	Missing	4 (11.1%)	1 (3.8%)	5 (8.1%)

Overall, 63% of respondents were male, 39% were aged between 55 and 64 years, and a further 34% were between 45 and 54 years of age. About 15% were employed at a statutory health insurance, 8% at the National Association of Statutory Health Insurance Physicians (Kassenärztliche Bundesvereinigung), 8% worked for a private health insurance, and a further 5% was employed at the Medical Review Board of the Statutory Health Insurance Funds (Medizinischer Dienst der Krankenkassen). Twenty-seven percent of respondents had no managerial responsibility, a further 27% had managerial responsibility for ≤ 10 employees, and 19% had managerial responsibility for > 50 employees. About half (55%) of respondents stated to use evidence synthesis for informational purposes. A further 19% of the respondents were involved in the preparation of evidence syntheses, and 15% of the respondents used evidence syntheses as a basis for decision-making. In both scenarios, participants’ characteristics in terms of sex and affiliation do not deviate significantly from the originally generated sample of 204 persons and thus, no participation bias is suspected.

The respondents completed a total of 504 pairwise comparisons in scenario 1 (preparation of rapid review within 20 working days). In the full model, the coefficients of level 2 were consistently classified between level 1 and level 3. However, distances from zero were not uniform for the attributes “number of reviewers during screening”, “types of HTA domains”, and “number of databases”, i.e., perfect linearity cannot be assumed for these attributes (see Sup. Table  3 and 4). This was accepted in favor of the more economical linear difference model. Results for the linear difference model show preferences for higher levels for “number of reviewers during data extraction”, followed by “number of reviewers during screening”, “full-text analysis”, “publication period to be considered”, and “types of HTA domains”. The attribute "number of databases" did not reach statistical significance (Table 3).

**Table 3:** Results of the linear difference model for scenario 1

Attribute	OR [95% CI]	***p*** value
**Number of reviewers during data extraction**	1.611 [1.306; 1.987]	< 0.0001
**Number of reviewers during screening**	1.484 [1.245; 1.770]	< 0.0001
**Full-text analysis**	1.475 [1.165; 1.869]	0.0013
**Publication period to be considered**	1.382 [1.099; 1.737]	0.0056
**Types of HTA domains**	1.225 [1.046; 1.434]	0.0117
**Number of databases**	1.022 [0.816; 1.281]	0.8469

The respondents completed a total of 364 pairwise comparisons in scenario 2 (preparation of rapid review within 12 months). Similar to scenario 1, the coefficients of level 2 were consistently classified between level 1 and level 3. Distances from zero were basically uniform with the exception of “number of databases”. Concerning the latter, level 1 and level 2 were essentially rated as equal. However, level 3 of “number of databases” was significantly different from zero. Results of the linear difference model showed preferences for higher levels for “number of reviewers during data extraction”, followed by “number of reviewers during screening”, “full-text analysis”, and “types of HTA domains”. The attributes “number of databases” and “publication period to be considered” did not reach statistical significance (Table 4).

**Table 4:** Results of the linear difference model for scenario 2

Attribute	OR [95% CI]	***p*** value
**Number of reviewers during data extraction**	2.218 [1.701; 2.893]	< 0.0001
**Number of reviewers during screening**	1.832 [1.474; 2.278]	< 0.0001
**Full-text analysis**	1.753 [1.241; 2.476]	0.0015
**Types of HTA domains**	1.539 [1.267; 1.869]	0.0001
**Number of databases**	1.223 [0.969; 1.544]	0.0906
**Publication period to be considered**	0.966 [0.787; 1.186]	0.7435

Results of the explorative subgroup analyses indicate that participants who are involved in the preparation of evidence syntheses show a strong preference for carrying out a full-text analysis (scenario 1 OR 3.452; 95% CI 1.382–8.624; scenario 2 OR 4.271; 95% CI 1.952–9.342). Full-text analysis was given a lower preference by respondents who did not participate in the preparation of evidence syntheses themselves (scenario 1 OR 1.431; 95% CI 1.090–1.879; scenario 2 OR 1.267; 95% CI 0.828–1.938, not statistically significant). Due to the rather small number of participants in the subgroups, the generalizability of these findings is very limited. The full results of the explorative subgroup analyses are shown in the supplementary material  (see Sup Table 5).

The final question of the online survey, dealing with 11 attributes of an evidence synthesis, was answered by 57 participants (see Sup Table 6). "Data extraction by 2 reviewers "was rated highest (mean 3.96; SD 1.068). Average scores are in close proximity and the highest prioritized attribute was rated only 0.58 points higher than the lowest one.

## Discussion

Literature on the overall effects of methodological shortcuts in rapid reviews is scarce and analyses of the impact of methodological shortcuts on review quality did not show clear results [20, 21]. A literature search limited, e.g., in terms of the number of databases presumably leads to a smaller number of studies being included in comparison to classical systematic reviews [22]. Thereby, risks for selection, retrieval, and publication bias can increase which can distort the results of a review [8, 12], thus potentially leading to wrong decisions or recommendations [14]. If screening and data extraction are performed by one person, errors might remain undetected. For example, a recently published trial shows that single-reviewer abstract screening missed 13% of relevant studies, while dual-reviewer abstract screening missed 3% of relevant studies [23]. Similarly, Taylor-Phillips et al. (2017) found that a basic rapid review approach involving a single reviewer led to important inaccuracies in data extraction when compared to a systematic review. However, an enhanced rapid review approach with a second reviewer checking 20% of titles/abstracts and data extraction performed better and, according to the authors, may be an appropriate tool to expeditiously assess evidence [24]. Finally, a lack of quality assessment of the included articles may limit the validity of a rapid review as a whole [2, 12].

The present study analyzes preferences of decision-makers in healthcare and people involved in the preparation of evidence syntheses. Attributes and levels for the DCE were derived from published guidance by analyzing a number of rapid review method papers and extracting the steps for conduct of the respective format. Thus, attributes and levels were based on established approaches in rapid review methodology. Also, the method of DCE seems to be a suitable approach in the present analysis since the preparation of rapid reviews is usually limited by financial and temporal resources. By the fact that the pairwise comparisons constrain trade-offs, the respondents are prevented from classifying all attributes as very important.

The two scenarios address different timeframes (20 working days/12 months) and therefore include different numbers and definitions of levels. Nevertheless, for both scenarios, performing the data extraction by two persons in conjunction with quality assurance is very relevant. Similarly, screening by two persons with consensus and a full-text analysis of the literature are of great importance for the respondents. In scenario 2, the inclusion of several domains (economic, ethical, social, legal, and organizational issues) shows a stronger preference than in scenario 1. It is conceivable that with the longer working time in scenario 2, participants ascribe importance to the consideration of several domains. However, the higher relevance of the additional domains to be included could have also been influenced by the fact that scenarios 1 and 2 deal with different topics. The attributes “searches in several databases” and “publication period to be considered” show comparatively low preferences in both scenarios.

Potential for improvement of existing formats can be derived from the fact that decision-makers clearly expressed preferences for formats in which the process of screening and data extraction was performed by two persons and specific quality standards were attained. Based on these preferences, financial and temporal savings should not be realized by reducing the number of people involved in screening and data extraction. According to the participants of this survey, preferable methodological shortcuts involve restrictions in the number of databases, a consideration of a smaller number of HTA domains, or a shorter publication period. Presumably, the absence of trade-offs is reflected in the answers to the final question on 11 attributes of an evidence synthesis. Even though the data extraction by two persons was also rated as most important, the other individual attributes were rated comparably high, so no clear results can be derived.

The present study has several limitations. First, due to the aggregation of the originally defined 11 attributes to six attributes, only a selection of attributes could be examined and thus, preferences could not be derived for all steps in the preparation of rapid reviews. However, this reduction of attributes was deemed necessary to enhance the feasibility of the DCE. Second, the rather small number of 36 persons participated in scenario 1, and only 26 persons participated in scenario 2. However, statistically significant findings could be reported for the majority of attributes in scenario 1 and scenario 2, indicating their relevance. Third, one could argue the usage of the linear difference model. Though some of the attributes do not show a perfect linear relationship, the order of the levels was still preserved, i.e. the lower and higher levels lie on different sides of the medium level throughout. As a ranking of attributes was the major goal of this analysis rather than an interpretation on level basis, it was decided to use the more parsimonious model. Fourth, our sample consisting of decision-makers and people involved in the development of evidence syntheses is not a representative sample in a statistical sense. Fifth, there is some evidence for a higher proportion of employees in statutory health insurance in scenario 1 than in scenario 2 and it cannot be ruled out that the results were influenced by these differences. This unequal distribution may be due to the fact that (i) these employees have felt an affinity to scenario 1 and (ii) that the link to the survey might have been shared among colleagues.

Finally, the fact that the three attributes “number of reviewers during data extraction”, “number of reviewers during screening”, and “full-text analysis” have a similarly strong preference in the two scenarios suggests a generalizability. However, it must be taken into account that the importance of the individual work steps in practice depends on the respective research question. For example, the benefit of including further databases might be small for certain research questions. In the case of very short-term inquiries, it might be useful to focus on the clinical domains of safety and efficacy, and the economic assessment may be conducted at a later stage. Furthermore, a possible link between the attributes should be considered. Although, as described, the attributes do not overlap as such, they are part of a sequence in which alterations might affect process steps occurring later in time. For example, there might be little point to restrict the number of databases, in order to ultimately examine a large number of domains. The analysis principally reflects the view of German users and developers of evidence syntheses.

## Conclusions

Concluding, the present paper shows that the method of DCE can be applied to determine preferences for methodological aspects of rapid reviews. Our findings that decision-makers and researchers preparing evidence syntheses clearly expressed preferences for certain quality standards related to the process of literature screening and data extraction provides important insights. Current methodological approaches with a reduced number of people involved in screening and data extraction should be critically evaluated. Especially in times of global public health crises, such as the COVID-19 pandemic, rapid reviews gain importance. It must be ensured that rapid reviews are of acceptable quality to maximize their credibility and impact. Future research needs to further explore the impact of certain methodological alterations in the conduct of rapid reviews with the ultimate aim to develop formats which fulfill decision-makers’ preferences and expectations regarding the validity of rapid reviews.

## Supplementary Information

**Additional file 1: Sup. Table 1.** List of organizations. **Sup. Table 2.** Attributes and levels for scenario 2. **Sup. Table 3.** Results of the full model for scenario 1. **Sup. Table 4.** Results of the full model for scenario 2. **Sup. Table 5.** Results of the subgroup analyses. **Sup. Table 6.** Results for the question on 11 attributes of an evidence synthesis. **Sup. Figure 1.** Exemplary choice task (translated).

## Data Availability

The datasets used and/or analyzed during the current study are available from the corresponding author on reasonable request.

## Abbreviations

HTA: Health technology assessment
DCE: Discrete choice experiment
OR: Odds ratio
CI: Confidence interval
SD: Standard deviation
ISPOR: International Society for Health Economics and Outcomes Research
