# Supplementary material for: Discrete choice experiment to determine preferences of decision-makers in healthcare for different formats of rapid reviews
Source: Syst Rev. 2021 Apr 20;10:121. doi: 10.1186/s13643-021-01647-z (PMC8057003; doi:10.1186/s13643-021-01647-z)

**Supplementary material:**

**Sup. Table 1: List of organizations**

| **Website** | **Name of organization** | **Name of guidance** |
| --- | --- | --- |
| *https://www.cochrane.org/* | Cochrane | Rapid Research Needs Appraisal |
| *https://euroscan.org/* | EuroScan | A toolkit for the identification and assessment of new and emerging health technologies |
| *https://www.inahta.org/* | INAHTA | HTA Core Model for Rapid Relative Assessment |
| *https://www.iqwig.de/* | IQWiG | Rapid Reports |
| [*https://www.dimdi.de/*](https://www.dimdi.de/) | DIMDI | Kurz-HTA |
| *https://austria.cochrane.org/de* | Cochrane Austria | EbM-Aerzteinformationszentrum |
| *https://aihta.at/* | LBI-HTA | Rapid Assessment |
| *https://www.nihr.ac.uk/* | NIHR | Horizon Scanning |
| *http://www.has-sante.fr/* | HAS | Rapid Assessment |
| *https://www.cadth.ca/* | CADTH | Rapid Response Reference Lists and Summary of Abstracts Reports, Summary with Critical Appraisal, Rapid Response Systematic Review and Meta-Analysis |
| [*https://www.adelaide.edu.au/*](https://www.adelaide.edu.au/)*ahta/* | AHTA | Horizon scanning |
| *https://campbellcollaboration.org/* | Campbell Collaboration | Policy brief |

**Sup. Table 2: Attributes and levels for scenario 2**

| **Attribute** | | **Level 1** | **Level 2** | **Level 3** |
| --- | --- | --- | --- | --- |
| **Database searches** | Number of databases | *Medline and another database* | *Medline + 2 further databases* | *Medline + 3 further databases* |
|  | Number of reviewers for screening | *1 reviewer* | *2 reviewers, no seeking for consensus* | *2 reviewers and seeking for consensus* |
|  | Publication period to be considered | *Last 5 years* | *Last 10 years* | *Unlimited* |
| **Data extraction** | Number of reviewers for data extraction | *1 reviewer, no quality assurance* | *2 reviewers, no quality assurance* | *2 reviewers and quality assurance* |
|  | Full-text analysis | *Full-text analysis for easily obtainable literature only* | *Full-text analysis* |  |
| **Extent** | Type of HTA-domains | *Safety, efficacy, economic aspects* | *Safety, efficacy, economic and ethical aspects* | *Safety, efficacy, economic, ethical, social, organizational and legal aspects* |

**Sup. Table 3: Results of the full model for scenario 1**

| **Attribute** | **Level** | **Coefficient** | **95% confidence interval coefficient** | |
| --- | --- | --- | --- | --- |
| **Number of reviewers during data extraction** | 1 | -0.3374 | -0.6178 | -0.0570 |
|  | 3 | 0.6612 | 0.3889 | 0.9334 |
| **Number of reviewers during screening** | 1 | -0.5468 | -0.7832 | -0.3104 |
|  | 3 | 0.1210 | -0.1040 | 0.3460 |
| **Full-text analysis** | 1 | -0.5317 | -0.7977 | -0.2656 |
|  | 3 | 0.4804 | 0.1946 | 0.7662 |
| **Publication period to be considered** | 1 | -0.6525 | -0.9710 | -0.3340 |
|  | 3 | 0.3244 | 0.0790 | 0.5698 |
| **Types of HTA domains** | 1 | -0.1864 | -0.3800 | 0.0072 |
|  | 3 | 0.1144 | -0.1077 | 0.3364 |
| **Number of databases** | 1 | -0.1778 | -0.4930 | 0.1375 |
|  | 3 | 0.1813 | -0.0640 | 0.4266 |

**Sup. Table 4: Results of the full model for scenario 2**

| **Attribute** | **Level** | **Coefficient** | **95% confidence interval coefficient** | |
| --- | --- | --- | --- | --- |
| **Number of reviewers during data extraction** | 1 | -0.8304 | -1.1519 | -0.5090 |
|  | 3 | 1.0534 | 0.6987 | 1.4081 |
| **Number of reviewers during screening** | 1 | -0.6269 | -0.8964 | -0.3575 |
|  | 3 | 0.7411 | 0.4755 | 1.0067 |
| **Full-text analysis** | 1 | -0.3281 | -0.5095 | -0.1468 |
| **Types of HTA domains** | 1 | -0.5083 | -0.7528 | -0.2637 |
|  | 3 | 0.4083 | 0.1734 | 0.6431 |
| **Number of databases** | 1 | -0.0359 | -0.3549 | 0.2831 |
|  | 3 | 0.2646 | 0.0101 | 0.5192 |
| **Publication period to be considered** | 1 | 0.0631 | -0.1745 | 0.3007 |
|  | 3 | -0.0224 | -0.2639 | 0.2192 |

**Sup. Table 5: Results of the subgroup analyses**

| **Subgroup characteristic** | **Attribute** | **OR** | **95% confidence interval OR** | | | **p-value** |
| --- | --- | --- | --- | --- | --- | --- |
| **Scenario 1** | | | | | | |
| **Age <55 years**  **(n=20)** | Publication period to be considered | 1.719 | 1.245 | 2.375 | 0.0010 | |
|  | Number of reviewers during extraction | 1.718 | 1.287 | 2.293 | 0.0002 | |
|  | Full-text analysis | 1.703 | 1.232 | 2.355 | 0.0013 | |
|  | Number of reviewers during screening | 1.659 | 1.300 | 2.117 | <0.0001 | |
|  | Types of HTA domains | 1.208 | 0.975 | 1.497 | 0.0844 | |
|  | Number of databases | 1.033 | 0.757 | 1.409 | 0.8386 | |
| **Age ≥55 years**  **(n=13)** | Number of reviewers during screening | 1.810 | 1.303 | 2.516 | 0.0004 | |
|  | Number of reviewers during extraction | 1.641 | 1.155 | 2.332 | 0.0057 | |
|  | Publication period to be considered | 1.405 | 0.934 | 2.114 | 0.1026 | |
|  | Full-text analysis | 1.359 | 0.902 | 2.049 | 0.1428 | |
|  | Types of HTA domains | 1.223 | 0.935 | 1.599 | 0.1423 | |
|  | Number of databases | 0.914 | 0.615 | 1.357 | 0.6550 | |
| **Managerial responsibility**  **(n=24)** | Number of reviewers during extraction | 1.982 | 1.513 | 2.597 | <0.0001 | |
|  | Number of reviewers during screening | 1.695 | 1.362 | 2.109 | <0.0001 | |
|  | Publication period to be considered | 1.624 | 1.214 | 2.173 | 0.0011 | |
|  | Full-text analysis | 1.491 | 1.113 | 1.996 | 0.0073 | |
|  | Types of HTA domains | 1.276 | 1.046 | 1.556 | 0.0162 | |
|  | Number of databases | 1.129 | 0.850 | 1.501 | 0.4011 | |
| **No managerial responsibility**  **(n=9)** | Full-text analysis | 1.611 | 0.976 | 2.660 | 0.0624 | |
|  | Number of reviewers during screening | 1.581 | 1.067 | 2.344 | 0.0225 | |
|  | Publication period to be considered | 1.305 | 0.807 | 2.112 | 0.2775 | |
|  | Number of reviewers during extraction | 1.214 | 0.806 | 1.828 | 0.3537 | |
|  | Types of HTA domains | 1.065 | 0.777 | 1.461 | 0.6953 | |
|  | Number of databases | 0.798 | 0.500 | 1.271 | 0.3419 | |
| **Participation in the development of evidence syntheses (n=4)** | Full-text analysis | 3.452 | 1.382 | 8.624 | 0.0080 | |
|  | Types of HTA domains | 1.558 | 0.932 | 2.604 | 0.0910 | |
|  | Number of databases | 1.129 | 0.547 | 2.333 | 0.7423 | |
|  | Number of reviewers during screening | 1.053 | 0.600 | 1.849 | 0.8568 | |
|  | Publication period to be considered | 1.011 | 0.488 | 2.093 | 0.9766 | |
|  | Number of reviewers during extraction | 0.986 | 0.503 | 1.933 | 0.9665 | |
| **No participation in the development of evidence syntheses (n=28)** | Number of reviewers during screening | 1.805 | 1.461 | 2.229 | <0.0001 | |
|  | Number of reviewers during extraction | 1.766 | 1.383 | 2.254 | <0.0001 | |
|  | Publication period to be considered | 1.637 | 1.245 | 2.152 | 0.0004 | |
|  | Full-text analysis | 1.431 | 1.090 | 1.879 | 0.0099 | |
|  | Types of HTA domains | 1.183 | 0.986 | 1.419 | 0.0702 | |
|  | Number of databases | 0.983 | 0.756 | 1.279 | 0.8996 | |
| **Scenario 2** | | | | | | |
| **Age <55 years (n=14)** | Number of reviewers during extraction | 2.620 | 1.817 | 3.778 | <0.0001 | |
|  | Full-text analysis | 2.157 | 1.260 | 3.693 | 0.0051 | |
|  | Number of reviewers during screening | 1.992 | 1.406 | 2.821 | 0.0001 | |
|  | Types of HTA domains | 1.424 | 1.047 | 1.936 | 0.0241 | |
|  | Number of databases | 1.359 | 0.937 | 1.972 | 0.1061 | |
|  | Publication period to be considered | 0.939 | 0.701 | 1.257 | 0.6708 | |
| **Age ≥55 years (n=12)** | Types of HTA domains | 1.760 | 1.341 | 2.311 | <0.0001 | |
|  | Number of reviewers during screening | 1.717 | 1.259 | 2.342 | 0.0006 | |
|  | Number of reviewers during extraction | 1.716 | 1.080 | 2.726 | 0.0223 | |
|  | Full-text analysis | 1.513 | 0.941 | 2.433 | 0.0873 | |
|  | Number of databases | 1.106 | 0.802 | 1.524 | 0.5387 | |
|  | Publication period to be considered | 0.963 | 0.690 | 1.342 | 0.8225 | |
| **Managerial responsibility**  **(n=18)** | Full-text analysis | 2.243 | 1.482 | 3.394 | 0.0001 | |
|  | Number of reviewers during extraction | 1.773 | 1.287 | 2.442 | 0.0005 | |
|  | Number of reviewers during screening | 1.688 | 1.308 | 2.179 | <0.0001 | |
|  | Types of HTA domains | 1.390 | 1.120 | 1.726 | 0.0028 | |
|  | Publication period to be considered | 1.226 | 0.957 | 1.570 | 0.1074 | |
|  | Number of databases | 1.220 | 0.938 | 1.587 | 0.1385 | |
| **No managerial responsibility**  **(n=8)** | Number of reviewers during extraction | 4.603 | 2.252 | 9.410 | <0.0001 | |
|  | Types of HTA domains | 2.750 | 1.490 | 5.074 | 0.0012 | |
|  | Number of reviewers during screening | 2.564 | 1.423 | 4.621 | 0.0017 | |
|  | Number of databases | 1.757 | 0.918 | 3.361 | 0.0886 | |
|  | Full-text analysis | 1.141 | 0.528 | 2.466 | 0.7377 | |
|  | Publication period to be considered | 0.621 | 0.394 | 0.980 | 0.0409 | |
| **Participation in the development of evidence syntheses (n=8)** | Full-text analysis | 4.271 | 1.952 | 9.342 | 0.0003 | |
|  | Number of reviewers during extraction | 2.742 | 1.664 | 4.520 | <0.0001 | |
|  | Number of reviewers during screening | 2.042 | 1.269 | 3.285 | 0.0033 | |
|  | Number of databases | 1.333 | 0.805 | 2.207 | 0.2639 | |
|  | Types of HTA domains | 1.208 | 0.801 | 1.822 | 0.3673 | |
|  | Publication period to be considered | 1.042 | 0.705 | 1.540 | 0.8347 | |
| **No participation in the development of evidence syntheses (n=17)** | Number of reviewers during extraction | 2.566 | 1.767 | 3.725 | <0.0001 | |
|  | Types of HTA domains | 2.106 | 1.606 | 2.761 | <0.0001 | |
|  | Number of reviewers during screening | 1.984 | 1.505 | 2.614 | <0.0001 | |
|  | Full-text analysis | 1.267 | 0.828 | 1.938 | 0.2759 | |
|  | Number of databases | 1.267 | 0.931 | 1.725 | 0.1323 | |
|  | Publication period to be considered | 0.895 | 0.682 | 1.174 | 0.4221 | |

**Sup. Table 6: Results for the question on 11 attributes of an evidence synthesis**

| **Attribute** | **n** | **Minimum** | **Maximum** | **Mean** | **Standard-deviation** |
| --- | --- | --- | --- | --- | --- |
| **Study extraction by 2 reviewers** | 57 | 1 | 5 | 3.96 | 1.068 |
| **Searches in several databases** | 56 | 1 | 5 | 3.88 | 1.129 |
| **Depiction of a flowchart** | 31 | 2 | 5 | 3.87 | 1.118 |
| **Quality assurance during study extraction** | 56 | 1 | 5 | 3.71 | 1.187 |
| **Database searches with a highly sensitive search strategy (many hits)** | 56 | 1 | 5 | 3.71 | 0.986 |
| **Full text analysis for the entire literature** | 56 | 1 | 5 | 3.71 | 1.057 |
| **Database searches in several languages (e.g. English and German)** | 57 | 1 | 5 | 3.53 | 1.241 |
| **Coverage of many HTA domains (e.g. economics, ethics, social issues ...)** | 56 | 1 | 5 | 3.5 | 1.176 |
| **Long publication period (e.g. the last 10 years)** | 56 | 1 | 5 | 3.48 | 1.079 |
| **Literature screening by 2 reviewers** | 56 | 1 | 5 | 3.41 | 1.141 |
| **Finding consensus in literature screening** | 56 | 1 | 5 | 3.38 | 1.071 |

**Sup. Figure 1: Exemplary choice task (translated)**


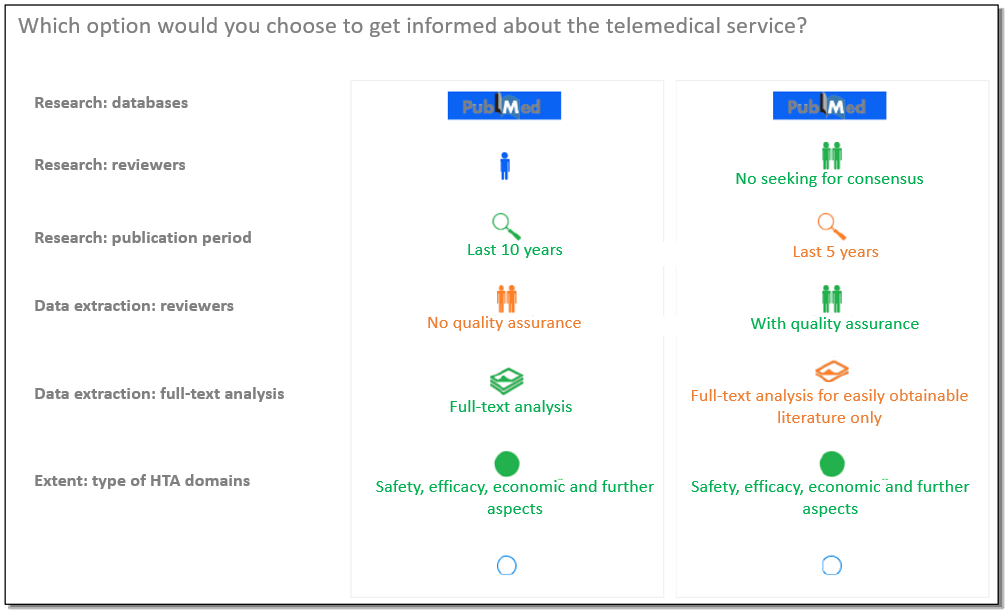

Supplement: Supplementary file 1 — Additional file 1: Sup. Table 1. List of organizations. Sup. Table 2. Attributes and levels for scenario 2. Sup. Table 3. Results of the full model for scenario 1. Sup. Table 4. Results of the full model for scenario 2. Sup. Table 5. Results of the subgroup analyses. Sup. Table 6. Results for the question on 11 attributes of an evidence synthesis. Sup. Figure 1. Exemplary choice task (translated). [file 13643_2021_1647_MOESM1_ESM.docx]
